# Inherited Retinal Diseases and Retinal Organoids as Preclinical Cell Models for Inherited Retinal Disease Research

**DOI:** 10.3390/genes15060705

**Published:** 2024-05-28

**Authors:** Kristen E. Ashworth, Jessica Weisbrod, Brian G. Ballios

**Affiliations:** 1 Institute of Medical Science, University of Toronto, Toronto, ON M5S 3H2, Canada; kristen.ashworth@mail.utoronto.ca; 2 Donald K. Johnson Eye Institute, Toronto Western Hospital, Toronto, ON M5T 2S8, Canada; jessweisbrod@icloud.com; 3 Department of Ophthalmology and Vision Sciences, University of Toronto, Toronto, ON M5T 3A9, Canada

**Keywords:** inherited retinal diseases, retinal organoid, gene therapy, stem cell therapy, neurodegeneration, retinitis pigmentosa

## Abstract

Inherited retinal diseases (IRDs) are a large group of genetically and clinically diverse blinding eye conditions that result in progressive and irreversible photoreceptor degeneration and vision loss. To date, no cures have been found, although strides toward treatments for specific IRDs have been made in recent years. To accelerate treatment discovery, retinal organoids provide an ideal human IRD model. This review aims to give background on the development and importance of retinal organoids for the human-based *in vitro* study of the retina and human retinogenesis and retinal pathologies. From there, we explore retinal pathologies in the context of IRDs and the current landscape of IRD treatment discovery. We discuss the usefulness of retinal organoids in this context (as a patient-derived cell model for IRDs) to precisely understand the pathogenesis and potential mechanisms behind a specific IRD-causing variant of interest. Finally, we discuss the importance and promise of retinal organoids in treatment discovery for IRDs, now and in the future.

## 1. Introduction

Inherited retinal diseases (IRDs) encompass an array of genetically and clinically diverse eye conditions that cause progressive vision loss due to gradual retinal cell degeneration. Variants in over 300 IRD-associated genes have been discovered, but cures have yet to be found. In recent years, gene and cell therapy have been brought to the forefront as potential IRD treatment opportunities for their ability to repair and replace degenerating retinal cells, with the goal of restoring vision. However, a fundamental challenge in advancing discovery in IRD treatments is a suitable, clinically translatable human model in which to test, screen, and evaluate therapy candidates. Animal models present multiple limitations; this is attributed to anatomical and electrophysiological differences between human and animal retinas, as well as the expression and function of homologous retinal genes. Retinal organoids—3D retinal structures grown from human pluripotent stem cells—present a favorable *in vitro* alternative. Retinal organoids recapitulate human retinal development, cell fate specification, maturation, and gene expression, and organoids can be derived from patients with specific IRDs [1].

In this review, we detail the genetic and clinical pathological bases for IRDs and why their complex and heterogeneous nature has presented challenges in treatment discovery. We then discuss the advantages and limitations of retinal organoids as a model of human retinogenesis and physiology compared to other preclinical models in use to study the complex pathogenesis of several IRDs. Finally, we review the model’s application to evaluate treatment candidates and potential cures for IRDs.

## 2. The Human Retina

### 2.1. Structure and Function of the Human Retina

The human retina is a complex and exquisitely organized central nervous system tissue located at the posterior aspect of the eye. The retina plays a key role in our ability to see, first by detecting visual information from the outside world, and then by passing on this information as electrochemical potentials to the brain for perceptual processing.

**Figure 1:**
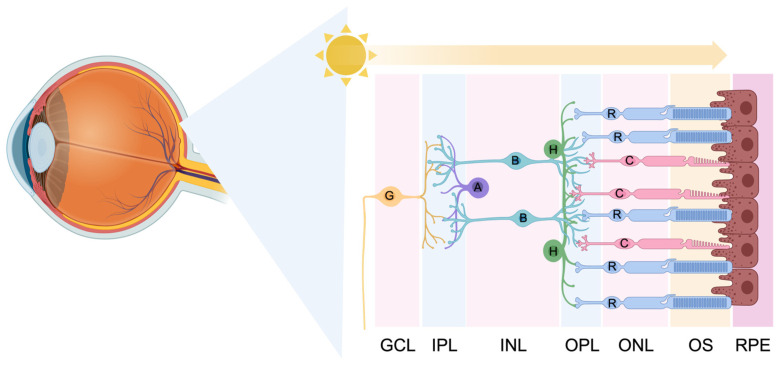
The layered organization of neuronal cells in a schematic cross-section of the human neuroepithelial retina. The neural retina has three cellular layers: the ganglion cell layer (GCL), the inner nuclear layer (INL), and the outer nuclear layer (ONL). In addition, there are two synaptic layers: the inner plexiform layer (IPL) and the outer plexiform layer (OPL). Other features and cell types within the retina include ganglion cells (G); amacrine cells (A); bipolar cells (B); horizontal cells (H); rod photoreceptors (R); cone photoreceptors (C); photoreceptor outer segments (OS); and retinal pigment epithelium (RPE). Graphic created with BioRender.com.

The neural retina (which excludes the retinal pigment epithelium (RPE)) is composed of three retinal nuclear layers (laminae) and two intermediary synaptic plexiform layers. Electrochemical signals are processed sequentially in these layers, prior to being transmitted to the brain. Rod and cone photoreceptors are light-sensitive retinal cells responsible for initiating most of the light response in the retina. Light passes through the eye and then the retina itself until it reaches the outer nuclear layer (ONL) of the retina where the rod and cone photoreceptors are located. In the outer segments, which extend from the cell bodies of the photoreceptors, incoming photons are absorbed by visual pigment molecules (such as Rhodopsin), and the phototransduction cascade is initiated. Graded membrane potentials are transmitted from the photoreceptors in the ONL and processed in the outer plexiform layer (OPL) through synaptic connections with cells in the inner nuclear layer (INL). The INL is composed of the cell bodies of bipolar, horizontal, amacrine, and interplexiform cells. These neurons modulate local signal processing in the retina before communicating through the inner plexiform layer (IPL) to the most proximal retinal layer, the ganglion cell layer. Retinal ganglion cells (RGCs) are the “output” neurons of the retina. RGCs receive signals from bipolar and amacrine cells, integrate and encode the visual information from surrounding receptive fields, and generate action potentials that are propagated through converging RGC axons to the optic nerve (Figure 1). Encoded visual signals leave the optic nerve to eventually be conveyed to, and interpreted by, the visual cortex [2].

### 2.2. Development of the Retina

The retina is a central nervous system structure, forming from the neural tube early in human embryogenesis [2]. Shortly after gastrulation, the eye field forms from the anterior neural plate. At embryonic day 32 (E32), the optic cup invaginates from the optic vesicles, which previously form from an embryonically specific “eye field” on the diencephalon. The proliferating inner layer of the optic cup gives rise to early multipotent retinal progenitor cells. These cells, which are actively proliferating, initiate the formation of the multi-layered neural retina through lamination, aided by a process known as “interkinetic nuclear migration” in the plane orthogonal to the expanding retinal area. Immature retinal progenitor cells give rise to all seven major post-mitotic cell types of the neural retina: ganglion cells, bipolar cells, amacrine cells, horizontal cells, Müller glial cells, and rod and cone photoreceptors. This process of post-mitotic differentiation begins at approximately fetal week 7 (Fwk 7), and the maturation of some retinal cells (including rod and cone photoreceptors) continues throughout the entirety of embryogenesis and into the human postnatal period [2,3].

The genesis of neural retinal cells in mammals is a sequential yet overlapping process, typically subdivided into two phases: early (~Fwk 7–18) and late (~Fwk 18–23) [3,4,5,6]. Cells in the early phase temporally differentiate in the following sequence: retinal ganglion cells, cone photoreceptors, amacrine cells, and horizontal cells [4]. Rod photoreceptor birth and development spans from midway through the early phase (~Fwk 12) into the late phase of fetal retinal development. Other cell types born in the late phase include bipolar cells and Müller glial cells [3,4,5]. Other mammals, such as mice, have a similar order of retinal cell genesis [5], suggesting conservation in the sequence of cell fate specification across species. By Fwk 14, the human retina has formed the three cellular layers (the ONL, INL, and GCL) and two synaptic layers (OPL and IPL) of the neural retina. At Fwk 20, the normal central human retina contains a mature population of retinal cells from all seven retinal cell types (with continued maturation in the rod photoreceptors postnatally) [6].

## 3. Inherited Retinal Diseases

### 3.1. Genetics of Inherited Retinal Diseases

**Figure 2:**
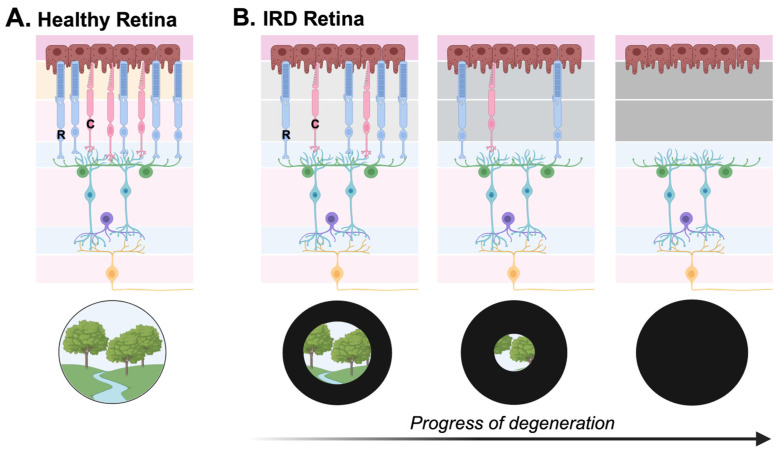
(**A**) A healthy human retina with an intact population of photoreceptors in the ONL. (**B**) A retina affected by IRD-causing pathogenic variant(s) with progressive degeneration of photoreceptor cells and/or RPE, with irreversible vision loss over time. Often, this vision loss occurs from the peripheral visual field inwards, as depicted in the lower panel of (**B**) (first with the loss of rod photoreceptors, R, then cone photoreceptors, C). Graphic created with BioRender.com.

IRDs are a genetically and clinically heterogeneous group of retinopathies that commonly result in the degeneration and loss of retinal photoreceptors. The result is irreversible visual impairment (Figure 2). It is estimated that approximately 5.5 million people are affected by IRDs worldwide [7], and although considered a group of rare diseases, they collectively remain a leading cause of retinal blindness [1,8].

The majority of IRDs are monogenic disorders that follow classic Mendelian patterns of inheritance: autosomal dominant, autosomal recessive, and X-linked. The list of IRD-associated genes is expansive and continuing to grow. To date, over 300 genes have been identified as harboring IRD-causing variants. However, pathogenic variants in some genes are more prevalently implicated as IRD-causative and include (but are not limited to) *RPGR*, *RHO*, *USH2A*, *ABCA4*, *EYS*, *CEP290*, and *MYO7A* [1,9]. Adding to this genetic complexity is the fact that variants within the same IRD-associated gene can produce a variety of different clinical phenotypes with respect to clinical retinal disease and degeneration [9,10].

### 3.2. Clinical Classifications of Inherited Retinal Diseases

IRDs have historically been categorized based on their clinical features, related to the structural changes and/or electrophysiologic dysfunction of the retina. There is considerable diversity in the clinical onset, symptoms, progression, and severity of different clinical IRD diagnoses.

For example, IRDs can be classified as early or late onset: symptoms can manifest during early childhood (e.g., Leber congenital amaurosis, achromatopsia, and Usher syndrome type 1), while other disorders do not present with symptoms until adolescence, or even later in life (e.g., Stargardt disease and some types of retinitis pigmentosa). In addition, IRDs can be categorized according to other clinical features, such as whether the retinopathy is non-syndromic (i.e., if only the retina is involved) or syndromic (i.e., if other extra-ophthalmic organs or systems are impacted) and/or if the disease is stationary or progressive. For progressive IRDs, the rate of progression and severity of vision loss can vary significantly from patient to patient, even among individuals and family members sharing the same familial variant(s) [9].

Though the molecular origins of IRDs are complex and unique to the functions of specific gene products, most IRD-causing mutations cause disruptions to the proper encoding of proteins involved in the structure and/or function of photoreceptors. Clinical phenotypes frequently converge, as is the case for the clinical diagnosis of retinitis pigmentosa (RP). Pathogenic variants in over 70 genes have been shown to cause the clinical phenotype of RP: since degeneration for most RP patients begins with the rod photoreceptors, clinical symptoms of night blindness and peripheral vision loss manifest first. Later, cone degeneration occurs, leading to progressive field narrowing, central vision loss, and, in a majority of cases, legal blindness [2,7].

Owing to the variety of clinical presentations and underlying genetic heterogeneity, IRDs are challenging to both diagnose at the bedside and study in the lab [9,10].

## 4. Current Approaches to IRD Treatment Discovery

Currently, there exists only one FDA-approved IRD treatment, Luxturna (voretigene neparvovec-rzyl), a gene therapy used to treat RPE cells in *RPE65*-associated retinal dystrophy [11]. The development of Luxturna represents a breakthrough in IRD treatment discovery and in molecular medicine in general; it was the first gene therapy approved for in vivo use in humans. However, significant headway is still required to uncover treatments for the multitude of remaining molecular causes of IRDs.

Broadly speaking, there are two main approaches that are being studied in preclinical IRD research: (1) to support, recover, or “fix” endogenous photoreceptors and/or other implicated retinal cell populations within the host retina; and (2) to replace these retinal cells, and/or their function, altogether.

### 4.1. Gene Therapy

Gene therapy is often aimed at addressing the first of these two approaches. In gene replacement therapy, healthy genetic material (DNA or RNA) is delivered to the diseased retina, usually by means of a viral vector. The goal is to replace the expression of the gene product and thereby restore visual function [12]. For those genetic causes of disease that are not related to loss-of-function or insufficiency, but rather related to dominant gain-of-function or aberrant splicing, gene-editing technologies (e.g., CRISPR/Cas9 editing) may provide an alternative approach, and clinical trials utilizing this technology are underway.

A notable example of this is EDIT-101, a CRISPR/Cas9 gene-editing therapy that was developed to treat *CEP290*-related IRDs caused by the c.2991+1655A>G mutation in intron 26 (IVS26) of the *CEP290* gene. This variant is the most frequent mutation in Leber congenital amaurosis type 10 (LCA10) patients. It is a deep intronic variant that generates a cryptic splice site, causing the downstream insertion of a pseudoexon in *CEP290* and producing a premature stop codon that leads to a truncated CEP290 protein. EDIT-101 works by deleting IVS26 from the mutant allele of photoreceptor cells in vivo after subretinal injection. A Phase 1/2 clinical trial was launched to assess the safety and efficacy of EDIT-101 in patients with *CEP290* disease. Recently, the trial produced exciting results, showing no serious adverse outcomes of the subretinal injection of EDIT-101; in addition, treatment with EDIT-101 produced meaningful improvements in multiple vision-related outcomes and assessments (including improved cone-mediated vision and best-corrected visual acuity by full-field stimulus testing and mobility testing) for at least half of the adult study cohorts in each test [13].

In the space of gene therapy, an alternative to gene-editing technologies are RNA-based therapies, which often involve variant silencing (e.g., in *CEP290*-associated Leber congenital amaurosis [14,15]) or exon skipping (e.g., in *USH2A*-associated retinitis pigmentosa [16]) and have shown promise in potentially addressing challenging variants or large genes not amenable to the current adeno-associated viral vector delivery for gene replacement therapy.

For example, another candidate developed to treat *CEP290*-associated LCA10 is sepofarsen, an RNA antisense oligonucleotide (AON) drug. Sepofarsen was tested in a Phase 1b/2 trial led by Russell et al. (2022). It targets the problematic *CEP290* c.2991+1655A>G mutation by binding to the deep intronic variant in the pseudoexon, preventing splice factors from recognizing it so that normal *CEP290* splicing can occur, and proper CEP290 protein formation is carried out. Sepofarsen was tested for safety and efficacy in a 12-month period by injecting it intravitreally into the worse-seeing eye of 11 patients with LCA10 at varying doses. The findings showed significant improvement in visual acuity and retinal sensitivity in the treated eye based on full-field stimulus testing and a mobility navigation challenge [15]. Following the success of this trial, a randomized, double-masked, sham-controlled Phase 3 study (ILLUMINATE) was conducted with 36 participants across 14 study locations in Europe, North America, and Latin America [17]. The trial was discontinued after the study failed to meet its primary and secondary endpoints: no significant improvement in visual acuity or improvement in mobility course navigation was demonstrated in the sepofarsen-treated participants compared to the sham-controlled participants [14].

Exon skipping as a therapeutic strategy to treat *USH2A*-associated IRDs has made significant headway in the past few years [16,17]. Designing a treatment that induces the skipping of exon 13 is of particular interest because this is the exon where two of the most common mutations in the *USH2A* gene, c.2299delG and c.2276G>T, are located. In 2021, Dulla et al. tested a therapeutic candidate that induced skipping of *ush2a* exon 13 in mutant zebrafish. They found that morpholino-induced exon 13 skipping resulted in the proper formation of the usherin protein and restored retinal function. Following these findings, the researchers used the human antisense oligonucleotide candidate, QR421a, to successfully induce exon 13 skipping *in vitro* in an iPSC-derived photoreceptor culture from a patient with a homozygous *USH2A* c.2299delG mutation. The analogous AON candidate for mice, mQR-421a, was then used to test the AON’s efficacy in vivo: after intravitreal injection, mQR-421a induced exon 13 skipping in mouse photoreceptor cells and demonstrated a long duration of action, with high levels of exon skipping still maintained 203 days post-injection [17]. Following this, a 24-month Phase 1b/2 clinical trial (the Stellar trial) was conducted to test the safety and tolerability of QR-421a in 20 patients with biallelic mutations in exon 13 of *USH2A.* Patients received a single dose of QR-421a (or sham) injected into one eye intravitreally. The findings showed that treatment with QR-421a was well tolerated and led to significantly improved retinal sensitivity compared to the untreated eye and sham controls [16]. Unfortunately, further evaluation of QR-421a with the ProQR Phase 3 clinical trial (SIRIUS) was halted in 2022.

Overall, in the last decade, there have been promising advances in the field of gene therapy research for specific types of IRDs. However, limitations to the clinical application of these gene-specific approaches exist. Given the hundreds of different genes associated with clinical IRDs, it is challenging to develop tailor-made therapies for each monogenic disorder. And, given the variety of molecular mechanisms of disease, there is also a significant challenge in the development of gene therapies that can effectively target a wide array of variant-specific phenotypes. In other words, it is difficult to develop a one-size-fits-many treatment [1,8,9,10,11,12]. In addition, gene therapy may not be the best strategy for patients with advanced-stage disease, where endogenous photoreceptors in the patient retina have degenerated, and thus the “substrate” for gene therapy is no longer present [9,11]. In these cases of advanced photoreceptor degeneration, the approaches of optogenetics are being used to introduce the expression of light-sensitive moieties into other retinal cell types (e.g., bipolar cells and/or retinal ganglion cells) to restore light sensitivity and vision to the retina [18,19,20].

### 4.2. Cell Therapy

Preclinical research for cell-based therapies in the retina is primarily aimed at developing cell replacement therapies, that is, to restore visual function by replacing the population of diseased cells in a host retina with transplanted donor cells. Cell therapy has the potential to aid individuals with advanced disease, where there has been extensive loss of photoreceptors [21]. The application of stem cell replacement therapy for IRD treatment will be further explored in Section 5.4.3.

### 4.3. Models for IRD Research

For decades, animal models (including mice, zebrafish, and chicks) have been used, by way of in vivo genetic and embryonic manipulation studies, and ex vivo studies with retinal explants, to investigate retinal development, pathogenesis, and mechanisms of IRDs. These models have also been used to test experimental IRD treatments [22,23,24] and thus have played a vital role in advancing IRD treatment discovery. However, they present multiple limitations in their application to human research: compared with humans, animals differ in terms of their retinal anatomy and function, retinal cell subpopulation composition, and their conservation of specific genetic sequences, all of which can cause differences in how gene-specific IRD phenotypes and patterns of degeneration manifest in animals versus humans [25,26]. For example, mice, one of the most prevalent in vivo models for IRD research, have vast anatomical differences in the retina compared to humans: for example, mice lack a macula—the central, cone-rich region of the human neural retina—which limits their use in studying IRD-associated cone and macular diseases [27]. Another common animal model that has been used for decades in in vivo IRD studies is zebrafish. Although this species better recapitulates human retinal anatomy compared to mice (namely, having a cone-rich central retina), it is limited in its similarities to humans in other respects: unlike humans, zebrafish have a remarkable capacity to regenerate damaged neurons. Therefore, the patterns and extent of neurodegeneration that can manifest in human IRD pathogenesis cannot be precisely reflected in this model [28].

Human stem-cell-derived models have emerged as an alternative to animal models of disease, offering important insight into human retinal development and disease. With increasing sophistication in the technologies supporting human stem cell culture, retinal cell models have been generated using human pluripotent stem cells (hPSCs) for the creation of two-dimensional culture models and three-dimensional organoid models. The hPSCs used for these models can be obtained from human embryonic stem cells (ESCs) or human induced pluripotent stem cells (iPSCs). In two-dimensional culture studies, iPSCs are differentiated into retinal cells on an adherent tissue culture plate [29,30]. The development of specific retinal cell types and cell-to-cell interactions in co-cultures can then be studied. For example, Hayashi et al. (2016) created 2D cultures derived from iPSCs that spontaneously formed a variety of ocular cell types, including neural retinal cells, amongst other ocular cell types (such as the RPE, the lens, and the ocular surface ectoderm). During the process of differentiation, the cells formed distinct, lineage-specific domains in the 2D culture that the researchers named “self-formed ectodermal autonomous multi-zones” (SEAMs). The 2D SEAM formation closely mimicked whole-eye development, giving temporal insight on the differentiation of ocular cells in human eye development. In addition, the researchers found that cells dissociated from the functional ocular surface epithelia zone had regenerative capacity once isolated from the 2D cultures, rendering them a potential therapeutic candidate for anterior eye transplantation [31].

Two-dimensional cell models present some limitations in studying neural retinal degeneration, primarily due to their inability to replicate the 3D complex layering and organization of the native neural retina and the intercellular and synaptic interactions between retinal cells of the ONL and INL. As such, another *in vitro* model that addresses these limitations has been developed: neural retinal organoids (hereafter referred to simply as “retinal organoids”, unless otherwise specified).

## 5. Retinal Organoids as a Preclinical Cell Model

Retinal organoids are three-dimensional retinal structures derived from pluripotent stem cells *in vitro.* Retinal organoids are often regarded as “miniature retinas”, attributed to their remarkable ability to organize into a 3D laminated, multi-layered structure akin to native neural retina, containing all seven major retinal cell types with some light-responsive functional characteristics, such as bright light response patterns similar to those seen in ON and OFF RGCs of the developing in vivo neural retina. In addition, human-derived retinal organoids capture aspects of human retinal physiology that animal-derived retinal organoids do not. The capacity to derive retinal organoids from patient stem cells (mutation-specific) renders them a powerful tool for investigating the mechanisms in which certain IRDs develop and how the cells composing the retina jointly respond to treatment candidates [32]. As such, retinal organoids are becoming a widely accepted and powerful preclinical cell model for IRD therapy discovery.

### 5.1. The Origin of Retinal Organoids

In 2011, Eiraku and colleagues made the landmark discovery that differentiating mouse embryonic stem cell aggregates had the unexpected quality of self-organizing into 3D optic cup-like neural retinal structures *in vitro.* Based on the manipulation of signaling pathways known to be important in retinal development in vivo, Eiraku developed a carefully controlled, stepwise set of culture conditions conducive to retinal differentiation. Unexpectedly, when grown in 3D aggregates, spontaneous optic cup-like structures would form in the culture. Shortly after, human neural retinal organoids were derived, first by the creation of retinal organoids from human embryonic stem cells [33] and then in human iPSCs [34]. Further optimization of human retinal organoid protocols improved the yield and survival [35,36,37] of organoids. As these techniques became established, investigators worked on creating retinal organoids to model various retinopathies, such as inherited retinal diseases, by using patient-derived iPSCs from patients with specific Mendelian retinal disorders [38,39,40,41]. By studying cell-to-cell interactions, cell integration, and retinal development in the context of a human 3D retinal model of disease, researchers have gained immense insight into the mechanisms in which specific treatments may be able to function therapeutically [41,42,43,44,45].

### 5.2. Growing Retinal Organoids from hPSCs

The generation of retinal organoids from hPSCs commonly involves using a 3D-to-2D-to-3D or 2D-to-3D culture differentiation method. In both approaches, stem cells are first differentiated into a neuroectoderm lineage, then a retinal fate through a sequence of growth factors and signaling molecules in various media and culture conditions [46].

In the 3D-to-2D-to-3D method, hPSCs are first suspended as aggregated single cells, and an embryoid body (EB) medium is used to promote EB formation. Once EBs are formed, the culture is switched to neural induction media (3D stage). After approximately one week, EB aggregates are transferred to an adherent plate to allow for neural cluster formation (2D stage). At approximately two to three weeks of neural differentiation (when optic vesicles begin to form), the culture is scraped and switched to 3D suspension with retinal differentiation media and maintained as retinal tissue begins to mature [47].

**Figure 3:**
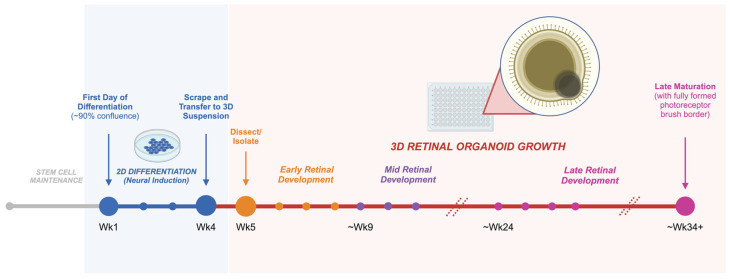
Timeline of differentiation for retinal organoids following a 2D-to-3D protocol, from stem cell stage to late maturation. Graphic created with BioRender.com.

An alternative approach is to differentiate cultures in a 2D-to-3D manner (Figure 3). Briefly, hPSCs are grown in 2D to 90% confluence on an adherent plate, at which point the culture medium is switched to induce neural induction. The 2D neural induction period occurs for three to four weeks before optic vesicles (what will be termed ‘retinal organoids’ when transferred to a 3D suspension) begin to form. Organoids are scraped from the 2D adherent plate and transferred to a 3D suspension. Subsequent to this, organoids are manually dissected and isolated into individual wells of a 96-well non-adherent plate for long-term maintenance with retinal maturation media containing fetal bovine serum, taurine, and retinoic acid [35,36]. Described in detail by Reichman et al. (2014), this protocol bypasses the first step of differentiation with EB aggregates, while forming neural retinal structures within two weeks of differentiation. This approach may be advantageous for its improved scalability compared to the 3D-to-2D-to-3D protocol: there are fewer steps involved, with less exogenous molecules required to be added to the media in the initial steps of differentiation. Overall, it can provide a simplified means to produce self-forming retinal organoids [35].

### 5.3. Recapitulating Human Retinal Development with Retinal Organoids

Human neural retinal organoid development *in vitro* has been demonstrated to closely parallel the sequence of in vivo human retinal development [34,48]. In a study by Zhong et al. (2014), retinal organoids were found to recapitulate the time course of retinal cell birth as seen in human embryos: first with the appearance of ganglion cells at week 5 of organoid development (with the ganglion cell layer being fully formed by week 12–13), and then the appearance of photoreceptors, along with the initial formation of the ONL, at week 7. By week 21, Rhodopsin was found to be expressed in the outer segments of photoreceptor cells, reflecting the maturation of rod photoreceptors. The bipolar layer—the last cell layer to develop in human retinal cell development—was also the last to develop in older retinal organoids, occurring after week 22 of differentiation. Further studies have shown that retinal organoids mimic the spatiotemporal development of the human retina’s multi-layered histoarchitecture and, as such, may provide precise insight on stages of retinal disease pathogenesis in a developing human retina [34].

### 5.4. Use of Retinal Organoids in IRD Research

#### 5.4.1. Retinal Organoids for the Discovery of Novel IRD Variants

The first identified human IRD-associated genes were *RHO* [49] and *CHM* [50] in 1990, for autosomal dominant retinitis pigmentosa and X-linked choroideremia, respectively. Since then, over 300 additional genes associated with IRDs have been discovered [1], with ongoing discovery of new variants [51]. However, estimates indicate that as many as 24% to 53% of IRD patients face a clinical diagnosis without a known causative gene [52]. Retinal organoids have proved helpful in the detection and identification of novel genes implicated in inherited retinopathies.

Bronstein et al. (2020) demonstrated the efficient use of retinal organoids for use in diagnostic testing of known genes and for use in the discovery of new pathogenic variants. They detected the expression of 254 known IRD genes in mature (160-day-old) patient iPSC-derived retinal organoids, and, notably, IRD gene expression levels were detected to a greater extent in the retinal organoid transcriptome analysis (similar to normal human retina) compared to the same analysis conducted using patient skin cells and blood cells (with only 188 genes and 130 genes expressed, respectively, and at much lower transcripts per million for each cell type compared to retinal organoids). The patients (siblings, *n* = 2), from whom the retinal organoids were derived, had a clinical IRD diagnosis without a known causative variant. The researchers were able to use and compare the RNA-sequencing data obtained from retinal organoids of the affected versus unaffected siblings to uncover a novel non-coding pathogenic variant in the *CNGB3* gene [53].

In another recent study exploring novel variant IRD phenotypes, Burnight et al. (2023) created retinal organoids from a large cohort of patients with retinopathy caused by mutations in the allelically diverse *ABCA4* gene. The researchers were able to elucidate the pathogenicity of a specific variant in *ABCA4* (IVS30 + 12321 A>G) and found that its level of impact on vision loss was associated with other identified deleterious mutations on the opposing allele [54].

The implications of these types of findings extend beyond providing an accurate genetic diagnosis to a handful of patients; they showcase the powerful, efficient, and highly sensitive use of retinal organoids in transcriptome analysis for novel IRD gene discovery and identification [53]. In addition, the use of retinal organoids for the identification of future novel IRD-causing variants will broaden the cohort of patients eligible for specific IRD gene therapies.

#### 5.4.2. Retinal Organoids to Study Variant-Specific IRD Pathogenesis and Disease Mechanisms

Over the past two decades, retinal organoids have been developed to model a variety of retinal diseases. The challenge remains characterizing the often-unique organoid-based phenotype at the morphological, molecular, and/or functional level.

*USH2A* is one of the most prevalent IRD-causing genes and is the most common cause of autosomal recessive RP. As a result of the mutation, the usherin protein (encoded by the *USH2A* gene and located at the connecting cilium for structural support of photoreceptors) does not properly form; this leads to the progressive loss of photoreceptors over time [55]. In addition to its commonality as an implicated IRD-causative gene, the large size of *USH2A* lends itself to a large assortment of associated variants and a spectrum of clinical phenotypes [42]. As such, generating accurate diagnoses and prognoses for patients with *USH2A*-related IRDs remains both relevant and challenging. Mice are not an ideal model to study *USH2A* disease development, since *Ush2a* mutant mice only display mild and late-onset photoreceptor degeneration [43,55]. Mutant *ush2a* zebrafish have been employed as an in vivo model to emulate human *USH2A* photoreceptor degeneration [43]. However, as a non-human model, they pose challenges with respect to their evolutionary distance from humans; in addition, the regenerative nature of the species makes it difficult to study the progression of *USH2A* disease as seen in humans [42,43].

Human retinal organoids provide an opportunity to address these challenges. Guo and colleagues (2019) and Sanjurjo-Soriano and colleagues (2023) have undertaken early efforts to identify an *USH2A* disease phenotype in retinal organoids [38,42]. Guo et al. (2019) generated retinal organoids from iPSCs of a patient with a pathogenic deletion mutation (c.9127_9129delTCC) in *USH2A*. They found that, compared to healthy controls, the *USH2A* organoids exhibited abnormal neuroretinal and RPE development from early stages of organoid growth, including decreased organoid size, lower expression of laminin, increased apoptosis, and degenerative photoreceptors [38]. Furthering the elucidation of *USH2A* phenotypes *in vitro*, Sanjurjo-Soriano et al. (2023) created retinal organoids derived from six patients with *USH2A*-associated RP (from three patients with non-syndromic RP and three patients with syndromic RP), each carrying different variants. They found that *USH2A* organoids derived from patients with non-syndromic RP had a cone-specific phenotype with significantly lower cone-specific marker expression (Arr3 and RG-opsin) compared to that of syndromic *USH2A* organoids and healthy controls at day 150; as well, *USH2A* non-syndromic RP organoids had significantly lower photoreceptor brush border length (i.e., photoreceptor inner and outer segment projection length from the outer border of the ONL) compared to syndromic *USH2A* organoids and age-matched controls. From this study, the researchers were able to demonstrate distinct phenotypes between variants at mature stages of organoid development, based on measuring *USH2A* expression levels, photoreceptor outer segment formation, cone-specific marker localization, and usherin protein expression [42]. However, it remains unclear whether these differences between *USH2A* disease organoids and healthy organoids are defects of photoreceptor development and maturation, or post-mitotic health and survival. In addition, since *USH2A*-IRDs cause photoreceptor degeneration throughout the adult lifetime, it will be critical in future studies to examine the progression of photoreceptor degeneration at multiple time points once retinal organoids reach full maturation (~Week 34 and beyond). This will enable us to better understand the patterns of degeneration comprehensively: for instance, comparing photoreceptor brush border length in *USH2A* versus healthy organoids over a span of time at a fully mature stage of retinal organoid growth (Weeks 30 to 42 of organoid growth) will elucidate temporally when the degeneration of the brush border starts to occur, and to what extent. From there, it can be better identified when treatment intervention would be most effective.

Another prevalent cause of early-onset retinal dystrophy is mutations in the ciliary protein-encoding gene *CEP290*. The CEP290 protein is located at the transition zone of photoreceptors, between the basal body and ciliary axoneme connecting the photoreceptor inner and outer segments. The protein assists in the transport of ciliary vesicles and proteins (such as ARL13B) along the length of the photoreceptor connecting the cilium; it also plays a role in outer segment formation. Like *USH2A*, pathogenic variants in *CEP290* are numerous and contribute to a heterogeneous array of ciliopathy-related clinical phenotypes. Two clinical disorders associated with *CEP290* gene mutations include syndromic retinopathy (Joubert Syndrome and Related Disorders, JSRD) and non-syndromic Leber congenital amaurosis (LCA). Mouse *Cep290* mutants have been used to recapitulate *CEP290*-retinopathy, but are limited in their ability to precisely link variant-specific *CEP290* diseases with specific human clinical phenotypes [39]. Shimada et al. (2017) derived retinal organoids from patients with *CEP290*-LCA to characterize the abnormal development of photoreceptor cilia in *CEP290* disease and compared this with ciliogenesis observed from *CEP290*-JSRD fibroblast cultures to delineate human phenotypes specific to each disease caused by *CEP290* mutations. They found that, at a mature stage of differentiation (day 200), *CEP290*-LCA organoids had underdeveloped cilia formation, similar to what was seen in the *CEP290*-JSRD fibroblasts [39].

Further to disease characterization, retinal organoids derived from mutation-specific patient iPSCs can help us gain a better understanding of functional impacts and cellular mechanisms behind an IRD’s pathogenesis; this, in turn, can inform treatment research. For example, Zhang and colleagues (2020) derived retinal organoids from two patients with compound heterozygous mutations in the rod–cone dystrophy-causing *CRB1* gene (c.1892A>G and c.2548G>A) and identified altered *CRB1* splicing patterns that lead to downstream impacts on protein function (producing a truncated protein with reduced function in the core Crumbs complex) [44]. In another study focused on the mechanisms behind IRD pathogenesis, Gao et al. (2020) generated retinal organoids from patients with late-onset RP harboring a *PDE6B* mutation and demonstrated *PDE6B* disease-specific impacts on synaptogenesis and connecting cilium formation within the diseased photoreceptors. They were able to identify the variant’s effect on cGMP hydrolysis, finding significantly higher cGMP levels in mature disease organoids compared to healthy controls, pointing to a potential mechanism for *PDE6B*-associated RP [40].

These are a few examples to illustrate the kind of insight that can be achieved with retinal organoid models of human IRDs that their animal counterparts may not be able to provide. Furthermore, it is worthwhile to note that retinal organoids can be derived from any iPSC-derived human source, allowing us to develop models for numerous and even exceptionally rare IRDs that might affect only a few families or individuals. With the advent of CRISPR/Cas9 gene-editing technology in the last decade, we have an easier ability to manipulate the causative variants or partners in the molecular interactome. Attributed to these advances, disease-specific iPSC lines can be derived *in vitro*, without the need for de novo isolation from human patients [45]. In principle, any IRD in existence can be modelled and studied in the context of human retinal organoids; rendering it an immensely powerful tool.

#### 5.4.3. Retinal Organoids for the Discovery of IRD Therapies

In addition to discovering new mechanisms of human retinal disease, retinal organoids are being used to develop and test potential IRD treatments. Disease-specific organoids can be easily manipulated in culture, and cellular and molecular assays can be used to analyze any potential rescue or response.

As mentioned above, gene and stem cell therapy are the primary research areas in disease-specific treatments for IRDs. Gene replacement therapy is focused on restoring retinal function while there is still a large enough pool of photoreceptor cells to “fix” and repair [12,56]. There are many clinical trials currently underway investigating gene therapy for specific IRDs, and retinal organoids are helping to assist in the preclinical development and assessment of these gene therapies before they are moved on to clinical trials. For example, a pioneering study by Deng et al. (2018) developed retinal organoids derived from three patients with *RPGR* mutations. Pathogenic variants in *RPGR* are the most common cause of X-linked RP. The researchers observed a significant pathogenic phenotype in *RPGR*-retinal organoids, which included abnormal photoreceptor morphology (such as a shortened cilium) and electrophysiological activity. The researchers then tested a targeted gene therapy by using CRISPR/Cas9 gene editing to correct the *RPGR* mutation in the patient-derived iPSC lines. In the *RPGR*-corrected iPSC-derived retinal organoids, they demonstrated a rescue effect of the gene correction on photoreceptor morphology and electrophysiology at mature stages of retinal organoid development [57].

In the last decade, retinal organoids have also provided the means to carry out high-throughput preclinical screening of adeno-associated virus (AAV) gene therapy candidates for IRDs. AAV-mediated gene repair is the most common vehicle for gene therapy in this context. The AAV carrying the therapeutic gene can be introduced into the retinal organoid through the culture media; from there, vector delivery and transduction efficiency can be monitored by carrying out immunostaining or quantitative PCR to investigate the expression of the therapeutic gene [56]. In a recent study by Sladen et al. (2024), the effect of AAV-*RPGR* (*RPGR*, retinitis pigmentosa GTPase regulator) gene repair on CRISPR/Cas9-generated *RPGR* knock-out retinal organoids was studied. The authors demonstrated efficient transduction of the retinal organoids and a successful restoration of *RPGR* mRNA and protein expression and localization to the photoreceptor connecting cilium that was comparable to healthy retinal organoids [45]. The findings from this study provide a further mechanistic understanding of AAV-*RPGR* repair that can be applied in clinical trials currently underway [58,59] for this gene replacement gene therapy.

Stem cell replacement as an IRD treatment may be helpful in the clinical context of advanced retinal disease, where little to no photoreceptor cells remain. Developing better methods of engrafting healthy donor cells in the retina may be useful. To date, there has been progress in the in vivo investigation of ESC, iPSC-RPE, and retinal progenitor cell transplantation to rescue disease phenotypes in multiple rodent models of retinal degeneration [60,61]. For example, Davis et al. (2017) showed the successful transplantation of RPE stem cells (RPESCs) into a rat model of RPE disease (using the Royal College of Surgeons (RCS) rat). They identified the precise stage of differentiation (at four weeks of maturation) in which RPESCs are most effective in rescuing vision in the host rat retina. Pointed out in the discussion of the study, and important to note for other in vivo rodent models used for the investigation of IRD cell therapies, is the translatability of findings to human disease. The RCS rat, for instance, has a rod-rich retina; therefore, it may not be helpful for studying the therapeutic potential of RPE or photoreceptor transplantation for patients with age-related macular degeneration (where cones are lost) [62].

Important headway on the mechanisms behind retinal cell–cell communication relevant to cell therapy has been expounded in recent years. In studies led by Ortin-Martinez and colleagues (2017; 2021), it was found that material transfer (MT)—which involves the intracellular exchange of mRNA, proteins, and mitochondria, through nanotube-like connections—occurs between donor and host cells in both *in vitro* (2D cell cultures) and in vivo models of retinal cell transplantation. The researchers showed that GFP was bidirectionally exchanged between donor and acceptor photoreceptor cells; that transplanted donor photoreceptors extend protrusions to transfer GFP to acceptor photoreceptors; and that this transfer is significantly increased when cells were exposed to a Rho kinase inhibitor (Rho being an inhibitor of neurite outgrowth). Although the *in vitro* work from these studies used 2D cultures, the critical insight gained about donor photoreceptor–host photoreceptor interactions and integration can be applied moving forward in the study of cell therapy mechanisms within a 3D retinal structure [63,64].

Researchers can use retinal organoids to study stem cell transplantation in a few different ways. First, by growing retinal organoids and obtaining and dissociating the organoid’s retinal cells for use in transplantation to animals in vivo, researchers might gain insights about donor cell integration, functionality, and therapeutic potential [65]. For example, Gasparini and colleagues (2022) obtained human photoreceptors from iPSC-derived retinal organoids and transplanted the cells into mice with cone degeneration. Their findings revealed a successful engraftment of the human photoreceptors and showed that donor–host cell interactions promoted the polarization and morphological development of photoreceptor outer segments, associated with improved photoreceptor function [65]. In another recent study, Ribeiro et al. (2021) demonstrated the functional rescue of cone photoreceptors in an *rd1* mouse model of retinal degeneration after treated mice received a transplantation of human cone photoreceptors from human-derived retinal organoids. The transplanted cones had nascent outer segment growth and made synaptic connections with host bipolar cells. Upon testing for visual function, treated mice demonstrated improved light-evoked behaviors compared to untreated, diseased mice [66].

Another application of retinal organoids for IRD-stem cell transplantation studies has been for the direct engraftment of cultured organoid tissue as a retinal sheet into rodent [67] and, later, human retinas [68]. Hirami et al. (2023) created clinical-grade, allogeneic iPSC-derived retinal organoids, dissected the retinal tissue, and transplanted the organoid sheet into one eye of each of two patients (both of whom had clinical RP). The researchers demonstrated that after two years, the grafts in both subjects were stable, with no serious adverse events, and the engrafted eye had better visual function (based on a full-field light stimulus and monitor tests) compared to the untreated eye for each patient [68]. These findings provide a promising step towards the use of retinal organoids for personalized medicine in cell replacement therapy for patients with IRDs. Other clinical efforts with cell transplantation have been underway for the past decade, with exciting headway [69,70]. For example, jCyte recently announced in February 2024 that they will be launching a Phase 3 clinical trial for their allogeneic cell therapy, jCell, to treat RP patients; this followed a successful Phase 2b trial in which 39% of RP patients receiving the jCell transplantation therapy showed improved visual acuity 12 months post-treatment [69].

### 5.5. Limitations of Retinal Organoids as a Preclinical Model

Despite the immense advantages that retinal organoids have provided for IRD research, the model has limitations. For example, retinal organoids lack a vascular or immune system. The microenvironment of the human retina, in which the vascular and immune system are apart, play a major role in retinal growth, maturation, and functionality. Consequently, without these structures, retinal organoids should be considered semi-physiologic models, as they do not yet incorporate the influence these systems have on the health and development of retinal cells [71]. To address these challenges, future research may aim to develop a retinal organoid system capable of mimicking the functions of vascular and/or immune systems, as is being conducted in other organoid models of human tissue. For example, Sun et al. (2023) has successfully developed blood-vessel-like structures fused to cerebral organoids [72]. Other researchers are now investigating the ability to similarly integrate vascular structures into retinal organoids. Busskamp and Sharma (2023) have conducted preliminary investigations by mixing endothelial cells with iPSCs before retinal organoid differentiation. They found that by 30 weeks, the endothelial cells formed a branched and networking “vascular system” in the inner core of growing retinal organoids and that these “vascularized” retinal organoids had improved retinal tissue size compared to non-vascularized organoids [73].

To further integrate retinal organoids in physiological and disease modelling is to explore their use in the development of “assembloids”, tissue-specific organoids attached together. In fact, investigation into assembloid design and functionality with retinal organoids has already been underway for a few years—Fligor and colleagues (2021) successfully created functional assembloids comprised of a retinal organoid attached synaptically to cortical and/or thalamic organoids. The researchers demonstrated that there was deep axonal outgrowth from the RGCs of the retinal organoid to the post-synaptic targets elsewhere in the neural assembloid upon stimulation from environmental cues. These findings demonstrate the exciting potential for further studies of RGC outgrowth and pathfinding using an assembloid system. In addition, in the future, the creation of retinal organoids as assembloids with other tissue types has the potential to inform studies on human visual system development and diseases [74].

While from one perspective an absence of other tissue systems presents a limitation, the exclusion of vasculature and the immune system in the organoid model may provide an ideal “reductionist” model of the retina, which can be helpful when seeking to study specific neural retinal cell-to-cell interactions and signaling mechanisms in isolation.

Another limitation of the retinal organoid model concerns the considerable amount of variability that arises between iPSC lines during the retinal organoid differentiation process, as highlighted by Cooke et al. (2023). In their study, they demonstrated significant differences in retinal differentiation propensity and early cell fate determination within retinal organoids derived from various iPSC lines. Variability in this respect can result in challenges with the consistency and reproducibility of findings [75]. However, with ongoing efforts to improve, refine, and streamline protocols, this issue can hopefully be mitigated over time.

## 6. Conclusions

Inherited retinal diseases are heterogenous genetic eye diseases that are currently incurable. Their vast and complex nature, through variability in both clinical and genetic phenotypes, poses a significant barrier to treatment discovery. The use of a suitable preclinical model in which to effectively evaluate IRD pathogenesis and treatments can potentiate the discovery of cures. Retinal organoids show distinct advantages over other preclinical models in accomplishing the goal of modelling human retinal development, disease, and response to IRD treatment candidates. Since the inception of this model over a decade ago, human retinal organoids have been optimized to produce all major retinal cell types in an organized, 3D-laminated structure that spatiotemporally recapitulates human retinal development and some elements of physiology. As highlighted in this review, their ability to be derived from patient cell lines has enabled widespread use of the model for IRD research. Retinal organoids have played a role in the identification of novel IRD-associated variants and the study of pathogenic mechanisms behind specific IRD-causative genes and variants from early to mature retinal development. The application of retinal organoids as a reliable, high-throughput platform to test and screen gene and cell therapy candidates in human tissue is one of the most exciting uses of the model now and in the future; it places us in a promising position to cure blinding genetic eye diseases in the years to come.
